# Screening Sonography Detection of Liver Masses: Assessing the Non-emergent Nature of Focal Nodular Hyperplasia

**DOI:** 10.7759/cureus.109321

**Published:** 2026-05-20

**Authors:** Sergio Miravent, Carolina A Guerreiro, Ricardo J Cordeiro, Carla M Gomes, Bruna S Vaz, Teresa L Figueiredo

**Affiliations:** 1 Basic Emergency Service of Vila Real de Santo António, Unidade Local de Saúde (ULS) Algarve, Vila Real de Santo António, PRT; 2 Medical Imaging and Radiotherapy, School of Health, University of Algarve, Faro, PRT; 3 Medical Dosimetry, Joaquim Chaves Saúde, Lisbon, PRT; 4 Levante Family Health Unit of Vila Real de Santo António, Unidade Local de Saúde (ULS) Algarve, Vila Real de Santo António, PRT; 5 Faculty of Medicine and Biomedical Sciences, University of Algarve, Faro, PRT; 6 Imaging for Primary Health Care, Unidade Local de Saúde (ULS) Algarve, Faro, PRT

**Keywords:** focal nodular hyperplasia, incidental findings, magnetic resonance imaging, triage, ultrasonography

## Abstract

The Basic Emergency Service (BES) in Portugal operates in peripheral healthcare units with limited technological resources, where the main goal is rapid patient triage and timely referral whenever necessary. In units located far from a reference hospital, screening ultrasound may support clinical decision-making during the initial assessment. However, with its increasing use, incidental detection of abnormalities unrelated to the presenting complaint has also become more frequent, including focal liver lesions. We report the case of a 28-year-old woman presenting to a BES with abdominal complaints, in whom a screening ultrasound identified a hepatic mass that was ultimately considered unrelated to the symptoms motivating the emergency visit. Although the lesion raised diagnostic interest, the sonographic findings did not suggest an immediately life-threatening hepatic condition, and the overall clinical context did not justify emergent transfer to the reference hospital solely because of this incidental finding. The patient was therefore managed according to the acute complaint and referred for outpatient imaging characterization. Dedicated liver imaging later showed features most consistent with focal nodular hyperplasia, which remained a probable radiologic diagnosis in the absence of histologic confirmation.

This case emphasizes an important challenge in peripheral emergency settings: sonographers performing screening ultrasound should be able to recognize findings that require urgent hospital referral and distinguish them from lesions that may be safely investigated in an ambulatory setting without compromising patient safety. The role of screening ultrasound in this context is not to establish a definitive pathological diagnosis, but rather to support clinical orientation, reduce unnecessary alarms, avoid inappropriate emergency referrals, and contribute to more efficient use of healthcare resources.

## Introduction

The Basic Emergency Service (BES) in Portugal consists of a peripheral network of small emergency units that provide first-line urgent care, initial assessment, and stabilization before referral to more differentiated hospitals whenever a higher level of care is required [1,2]. In these units, the main objective is not exhaustive diagnostic workup, but efficient triage, early risk stratification, and appropriate referral according to the clinical context. In one Portuguese BES, screening ultrasound performed by sonographers has already been explored as a pilot workflow to support clinical decision-making in selected urgent presentations, particularly in settings located far from reference centers [3].

As ultrasound has become increasingly used in screening and triage settings, abnormalities unrelated to the presenting complaint may also be identified incidentally [4]. This is particularly relevant for focal liver lesions, which are commonly encountered on abdominal imaging and often present as asymptomatic or incidental findings [5,6]. Their spectrum is broad and includes both benign and malignant entities, creating a practical challenge in peripheral emergency settings: the operator must be able to identify the presence of a liver lesion, describe any potentially concerning sonographic features, and alert the treating physician so that the need for hospital referral or outpatient investigation can be assessed appropriately [7,8].

Focal nodular hyperplasia (FNH) is a benign liver lesion thought to arise from localized vascular abnormalities and typically does not require urgent treatment when characteristic imaging features are present.

Screening ultrasound refers to a focused, rapid examination aimed at detecting major abnormalities and guiding immediate clinical decisions, rather than providing full diagnostic characterization [9]. Its role is not to establish the exact pathological nature of a hepatic lesion (HL), but to support clinical decision-making, reduce unnecessary alarm, and avoid inappropriate emergency referral. Definitive characterization of focal liver lesions belongs to dedicated diagnostic pathways led by the appropriate specialties and may require integration of clinical history, laboratory findings, and contrast-enhanced cross-sectional imaging, with histopathological assessment reserved for selected cases [10,11]. Current recommendations indicate that indeterminate liver lesions detected on ultrasound generally require further characterization with contrast-enhanced magnetic resonance imaging, multiphasic computed tomography and contrast-enhanced ultrasound, whereas biopsy is not the first step in many scenarios [12,13].

Magnetic resonance imaging (MRI) has a particularly important role in this setting because its multiparametric approach allows non-invasive tissue characterization and improves differentiation between benign and malignant liver lesions. Conventional sequences, diffusion-weighted imaging, and dynamic contrast-enhanced evaluation are central to lesion assessment, while hepatobiliary phase imaging may add further value when liver-specific contrast agents are used. Even so, imaging findings must be interpreted within the broader clinical context, and diagnostic certainty may still require multidisciplinary discussion [14,15].

This case report demonstrates that in peripheral emergency settings, incidental imaging findings can be safely managed through suitable triage, without requiring an immediate definitive diagnosis. 

## Case presentation

A 28-year-old female patient presented to the BES with abdominal pain for two days and one episode of vomiting on the day of presentation.

On arrival at the BES, the patient was triaged as green according to the Manchester Triage System, indicating a less urgent condition. She reported abdominal pain rated 4/10 and mentioned a family history of cholecystitis in her grandmother. There was no documented history of oral contraceptive use, significant alcohol intake, estrogen exposure, or known previous liver disease. Her temperature was 36.4°C, blood pressure 105/67 mmHg, and pulse 74 bpm. Physical examination showed epigastric tenderness and a doubtful Murphy’s sign in the right upper quadrant (RUQ).

Given the epigastric/RUQ pain, an equivocal Murphy’s sign, and the clinical possibility of biliary disease, the physician requested a screening ultrasound and basic blood tests; the reported family history provided additional context. Basic blood tests showed no significant abnormalities. Video 1 shows partial abdominal screening ultrasound, in which axial liver sweeps with Doppler demonstrate a HL of heterogeneous echogenicity. 

**Video 1:** Axial ultrasound sweeps of the hepatic lesion Panel A: Subcostal ultrasound cine clip showing a cranially located heterogeneous hepatic lesion near the suprahepatic venous confluence and heart, positioned between the MHV and LHV with mild mass effect on the LHV. The lesion appears relatively well-defined and mildly heterogeneous. Panel B: Transverse Color Doppler view of the same lesion, again located between the MHV and LHV, showing mild mass effect on the LHV and limited intralesional Doppler signal, with a possible central traversing vessel. H.L., hepatic lesion; RHV, right hepatic vein; MHV, middle hepatic vein; LHV, left hepatic vein; AO, aorta; IVC, inferior vena cava; RA, right atrium; RV, right ventricle; LV, left ventricle.

**Figure 1:**
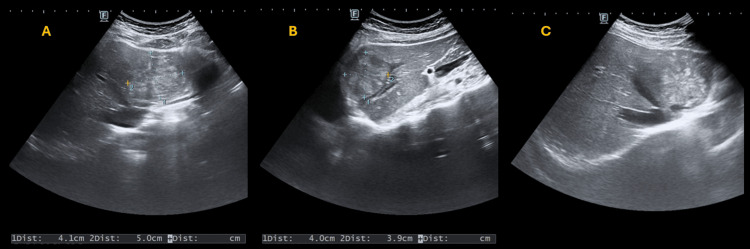
Approximate measurements and echogenicity of the hepatic lesion Panels A–C represent B-mode still images of  the hepatic lesion obtained in different planes. Panels A and B show biplanar measurements of approximately 4.1 × 5.0 cm and 4.0 × 3.9 cm, respectively. The lesion appears relatively well defined and mildly heterogeneous. In all panels, the contrast in echogenicity suggests a possible central scar-like area within the nodular lesion. Panel C provides an additional view of the lesion and its internal echotexture.

Video 2 shows longitudinal planes of the same lesion and its Doppler characteristics. The approximate dimensions of the lesion are presented in Figure 1. No abnormal findings were identified in the pancreas, gallbladder, or portal triad. After analgesic and antiemetic treatment, the patient improved rapidly. Since the symptoms that prompted presentation had resolved, the physician considered the hepatic finding unrelated to the acute presentation. The patient was referred to her family physician for further outpatient evaluation.

**Video 2:** Longitudinal ultrasound sweeps of the hepatic lesion Panel A: Longitudinal ultrasound cine clip of the liver showing a cranially located solid H.L. in close proximity to the heart, aorta, and IVC. During the sweep, the lesion appears relatively well circumscribed and mildly heterogeneous, with a small central anechoic focus visible in some frames. The clip highlights the close anatomic relationship of the lesion to the suprahepatic region and adjacent vascular structures, without definite evidence of gross vascular invasion in this video. Panel B: Longitudinal Color Doppler ultrasound view of the H.L., showing a relatively well-circumscribed solid lesion with scant internal Doppler signal and a possible central traversing vessel. Part of the color signal adjacent to the lesion likely corresponds to nearby vascular/cardiac flow rather than unequivocal diffuse intralesional vascularity. H.L., hepatic lesion; IVC, inferior vena cava; AO, aorta; IVC, inferior vena cava.

During outpatient radiology follow-up, a conventional ultrasound performed by a radiologist described a “well-circumscribed HL with heterogeneous echogenicity, measuring 5.35 × 4.28 cm; probably benign; conventional contrast-enhanced liver MRI recommended for further characterization”. The MRI findings are summarized in Video 3 using six representative excerpts from selected sequences and dynamic post-contrast phases that depict the lesion’s most relevant imaging features, including T1 hypointensity, mild T2 hyperintensity, intense arterial hyperenhancement, more homogeneous enhancement with relative near-isointensity on the portal venous and delayed phases, and a possible central scar with slight delayed enhancement. The final MRI report stated that a “well-defined solid nodular lesion is seen, with features compatible with FNH, measuring approximately 43 × 40 mm.” Overall, the entire diagnostic workup was conducted on an outpatient basis and favored a probable diagnosis of FNH. Follow-up surveillance was recommended at six months and, if the lesion remained stable, annually thereafter. 

**Video 3:** MRI findings summarized in panels A-F Panel A. T1 out-of-phase image showing the lesion as hypointense. Panel B. T2 SPIR image showing the lesion as mildly hyperintense. Panel C. T2 TSE image showing slight hyperintensity relative to the surrounding liver parenchyma. Panel D. Arterial phase image showing intense arterial hyperenhancement of the lesion. Panel E. Portal venous phase image showing more homogeneous enhancement with relative near-isointensity. Panel F. Delayed phase image showing relative near-isointensity of the lesion and slight enhancement of a possible central scar.

**Figure 2:**
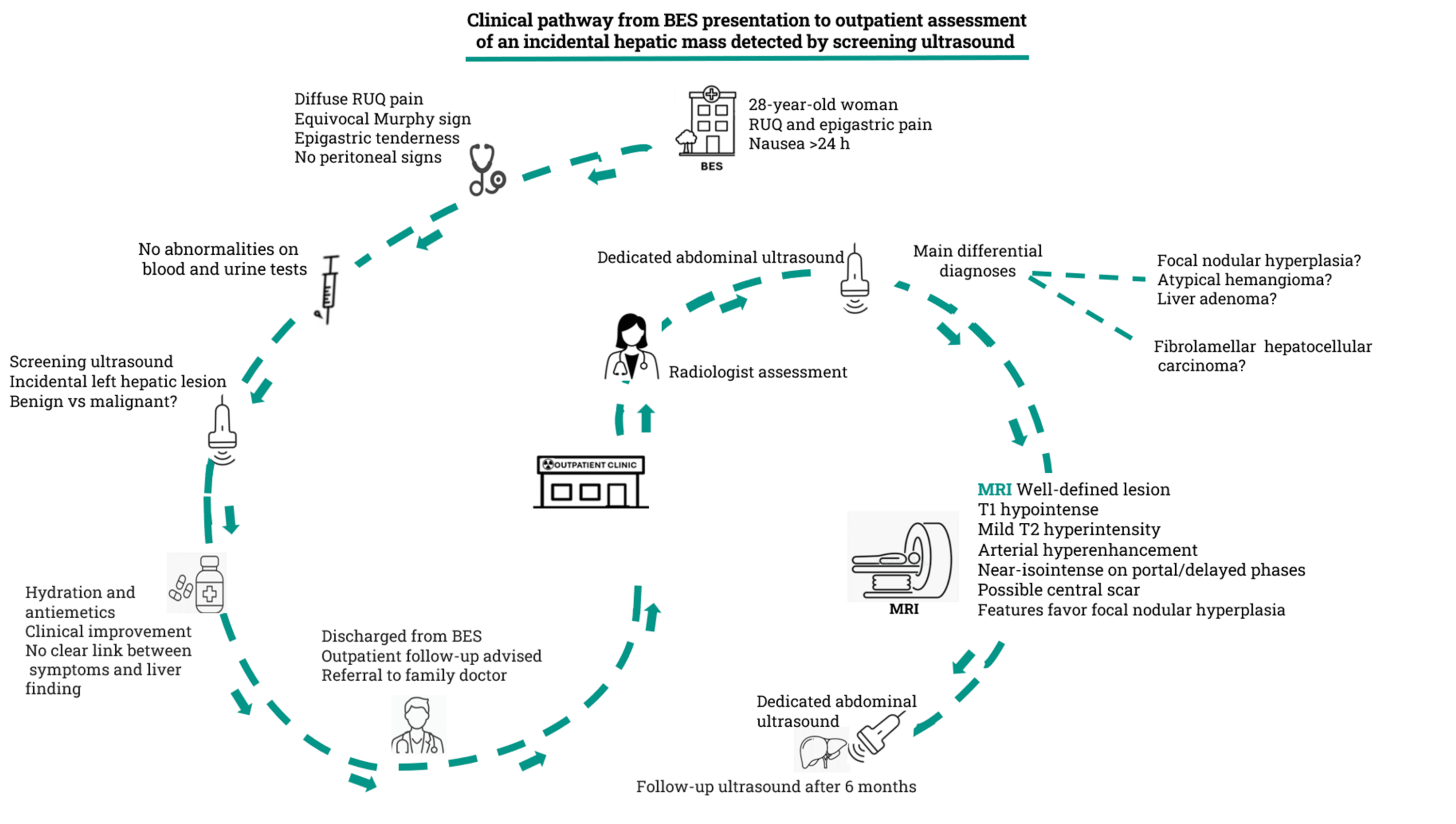
Timeline of the case and diagnostic follow-up This figure was created using Microsoft PowerPoint with non-generative icons sourced from publicly available medical illustration databases BES, Basic Emergency Service

Figure 2 presents a timeline of events to facilitate understanding of the sequential course of the case.

## Discussion

Benign focal liver lesions are commonly incidental findings, with hemangioma and FNH among the most frequent benign entities [16]. However, because imaging features may overlap among focal hepatic lesions, the differential diagnosis may also include hepatic adenoma and, more rarely, fibrolamellar carcinoma, particularly in lesions with a central scar [17-19].

Ultrasound alone is not sufficient for definitive lesion characterization, and operators performing screening examinations should recognize their limitations, avoid overinterpretation, and recommend further imaging when appropriate [20].

In this setting, the clinical dilemma is not only whether a focal liver lesion may ultimately be benign or malignant, but whether its imaging appearance and clinical context suggest an immediate risk to the patient. Similar incidental hepatic findings would warrant urgent referral if accompanied by hemodynamic instability, persistent or worsening abdominal pain, fever or sepsis, jaundice, significant laboratory abnormalities, free intra-abdominal fluid, suspected hemorrhage, abscess, biliary obstruction, vascular invasion, or a known oncologic background. In the absence of such features, outpatient characterization may be appropriate, provided that the finding is clearly communicated and follow-up is ensured.

In the present case, the incidental hepatic lesion did not justify emergent referral because the patient remained clinically stable, laboratory findings were unremarkable, and the acute symptoms improved rapidly with symptomatic treatment. This illustrates an important point in peripheral emergency units: not every abnormality detected on screening ultrasound is related to the presenting complaint or requires immediate escalation of care. When no signs of instability or immediately concerning features are present, ambulatory investigation may represent a safe and proportionate strategy. In this case, subsequent dedicated ultrasound and MRI supported a probable diagnosis of FNH, with MRI showing a well-defined hepatic lesion with low T1 signal, slight T2 hyperintensity, intense arterial hyperenhancement, relative return to near-isointensity on portal venous and delayed phases, and a possible central scar with slight delayed enhancement [21,22]. However, because histopathologic confirmation was not obtained, FNH should be regarded as a probable radiologic diagnosis in this case.

A slight discrepancy in lesion measurements between the screening ultrasound, conventional ultrasound, and MRI was noted, which is not unusual across operators, imaging planes, and imaging modalities.

## Conclusions

This case illustrates that incidental hepatic masses detected during screening ultrasound in peripheral emergency settings such as a BES are not necessarily related to the patient’s acute complaint and do not automatically warrant emergent referral. In this patient, clinical stability, unremarkable laboratory findings, symptom improvement, and the absence of immediately concerning sonographic features supported a proportionate outpatient diagnostic pathway rather than urgent transfer solely because of the incidental hepatic lesion. The patient was appropriately referred for dedicated imaging characterization of the finding, which supported FNH as a probable radiologic diagnosis, although histopathologic confirmation was not obtained. In this context, trained sonographers, working within a medically supervised, team-based workflow and within the limits of their professional scope, may contribute to early detection and clinical orientation by helping identify findings that require urgent escalation and distinguish them from those that can be safely investigated in an ambulatory setting, thereby supporting patient safety and more efficient use of referral pathways and healthcare resources.
